# Mortality and cancer in eosinophilic gastrointestinal disorders distal to the esophagus: nationwide cohort study 1990–2017

**DOI:** 10.1007/s00535-022-01904-5

**Published:** 2022-07-19

**Authors:** Adam C. Bledsoe, John J. Garber, Weimin Ye, Bjorn Roelstraete, Joseph A. Murray, Jonas F. Ludvigsson

**Affiliations:** 1 Department of Internal Medicine, University of South Dakota Sanford School of Medicine, Sioux Falls, SD USA; 2 Gastrointestinal Unit, Massachusetts General Hospital, Harvard Medical School, Boston, MA USA; 3 Department of Medical Epidemiology and Biostatistics, Karolinska Institutet, Stockholm, Sweden; 4 Celiac Disease Program, Division of Gastroenterology and Hepatology, Mayo Clinic, Rochester, MN USA; 5 Department of Pediatrics, Örebro University Hospital, Örebro, Sweden; 6 Division of Epidemiology and Public Health, School of Medicine, University of Nottingham, Clinical Sciences Building 2, City Hospital, Nottingham, UK; 7 Celiac Disease Center, Department of Medicine, Columbia University College of Physicians and Surgeons, New York, NY USA

**Keywords:** Eosinophilic gastritis, Eosinophilic gastroenteritis, Eosinophilic colitis, Eosinophilic gastrointestinal disorder

## Abstract

**Background:**

Eosinophilic gastrointestinal disorders (EGIDs) include inflammatory conditions with enteric infiltration of eosinophils and resulting symptoms. This study aims to examine a population-based sample of patients for prevalence, mortality, and cancer risk in EGIDs distal to the esophagus.

**Methods:**

Nationwide, population-based cohort study. EGID was identified through relevant biopsy codes from Sweden’s all 28 pathology departments through the ESPRESSO cohort. Individuals with EGID were then matched to general population reference individuals with similar age and sex. Study participants were linked to Swedish healthcare registers. Through Cox regression, we calculated adjusted hazard ratios (aHRs) adjusting for sex, age, county, calendar period, and education.

**Results:**

In total, 2429 patients (56% female) were found to have EGID distal to the esophagus, representing a prevalence of about 1/4800 in the Swedish population. Mean age was 44 years with 11% children at the time of diagnosis. Mortality was increased 17% in patients with EGIDs compared to reference individuals (aHR = 1.17; 95%CI = 1.04–1.33). Excess mortality was seen in gastric and small bowel eosinophilic disease, but not colonic disease (aHR = 1.81; 95%CI = 1.32–2.48, aHR = 1.50; 95%CI = 1.18–1.89, and aHR = 0.99; 95%CI = 0.85–1.16, respectively). Cause specific mortality was driven by cancer-related death (aHR = 1.33; 95%CI = 1.05–1.69). However, this study failed to show an increase in incident cancers (aHR = 1.14; 95%CI = 0.96–1.35). Comparison of EGID individuals with their siblings yielded similar aHRs.

**Conclusions:**

This study found an increased risk of death in patients with EGIDs distal to the esophagus, with cancer death driving the increase. Proximal gut disease seems to confer the greatest risk. There was no increase in incident cancers.

**Supplementary Information:**

The online version contains supplementary material available at 10.1007/s00535-022-01904-5.

## Introduction

Eosinophilic gastrointestinal disorders (EGIDs) include inflammatory disorders with increased eosinophilic infiltration of the target organ and resultant symptoms [1–3]. These include eosinophilic esophagitis (EoE), eosinophilic gastritis, eosinophilic gastroenteritis, and eosinophilic colitis. Signs, symptoms, and endoscopic findings vary depending on the organ involved and can include dysphagia, strictures, nausea and vomiting, abdominal pain, ulcers, iron deficiency, diarrhea, bloating, and protein losing enteropathy [4–6]. Deeper infiltration in the muscular or serosal layers can result in pain and ascites [7]. While the natural history of esophageal disease is more understood, data are limited for EGIDs distal to the esophagus.

Insurance database research estimates the prevalence of EGIDs distal to the esophagus at 6.3/100,000 for eosinophilic gastritis, 8.4/100,000 for eosinophilic gastroenteritis and 3.3/100,000 for eosinophilic colitis [6]. Survey-based estimates in the U.S. suggest a higher prevalence of 28/100,000 for EGIDs distal to the esophagus [3], while others suggested a prevalence of 5.1/100,000 for eosinophilic gastritis and 2.1/100,000 for eosinophilic colitis, utilizing aggregated health record data in the U.S. [8] A recent meta-analysis estimated a high prevalence of EGID distal to the esophagus of 1.9% of those presenting symptomatically for endoscopy [9]. EGIDs can be seen at any age but are most common in the fourth and fifth decades of life with a slight male predominance. However, studies on sex association have produced conflicting results [3, 4, 6, 7, 10].

Diagnosis is based on clinical history, enteric biopsy, and exclusion of secondary causes of eosinophilia. Eosinophils are normally found in the gastrointestinal (GI) tract distal to the esophagus. Accepted pathologic thresholds have been described and utilized in EGID trials [11, 12].

While a number of studies have explored mortality in EoE [13, 14], to our knowledge, there are no data on mortality, cancer risk and natural history in patients with EGID distal to the esophagus. The aim of this study is to describe mortality and cancer risk in that population, utilizing a population-based cohort.

## Methods

### The ESPRESSO cohort

In 2015 and 2017, all 28 pathology departments in Sweden were contacted to obtain histopathology report data from the GI tract [15]. These form the Epidemiology Strengthened by histoPathology Reports in Sweden (ESPRESSO cohort) from which all EGID cases were identified. EGID data were then linked to various healthcare registers that allowed us to examine cancer risk and mortality in EGID.

### Study population

#### EGID

We defined EGID as having a relevant SnoMed code indicating GI eosinophilia (M4715) distal to the esophagus (stomach to colorectum: topography codes T63-68, appendix (T66) not included). Data were restricted to 1990–2017 since the awareness of eosinophilic disorders was low before the 1990s. We did not include EoE since we have recently examined mortality in this disease entity (no association: HR = 0.97; 95%CI = 0.67–1.40)[14].

EGID was further divided into eosinophilic gastritis (T63), gastroenteritis (T64-65 duodenum through ileum), and colitis (T67-68) as suggested by Gonsalves [1]. Location of initial biopsy with eosinophilia (stomach, small bowel, or colon) was used to categorize as gastritis versus gastroenteritis versus colitis (Supplemental Table 1). Patients were categorized based on initial biopsy but allowed to develop subsequent EGID diagnosis in a different anatomic location.

To increase specificity for EGID we excluded any individual with an international classification of disease (ICD) code 9–10 for eosinophilia that may be due to hypereosinophilic syndrome/eosinophilic leukemia, lung eosinophilia/Loeffler syndrome, parasite infection, eosinophilic granuloma/Churg-Strauss, Histiocytosis X, eosinophilic meningitis, eosinophilic myocarditis, hereditary eosinophilia, or DRESS syndrome (Supplemental Tables 2, 3). To rule out that eosinophilia secondary to any cancer investigation, individuals with a record of GI adenocarcinoma in the last 12 months were also excluded (Supplemental Table 1). Finally, we excluded individuals where administrative reasons suggested they may not be in Sweden. The same exclusion criteria were applied also to reference individuals to rule out that any control individual would have adenocarcinoma or the above eosinophilic conditions when matched. For more details we refer to our review on the ESPRESSO study [15].

#### Reference individuals

The government agency *Statistics Sweden* matched each individual with EGID with ≤ 5 reference individuals on age, sex, county of residence, and year of birth (*n* = 11,719) from the Swedish Total Population Register (TPR) [16]. Reference individuals had no record of EGID at or before matching date but were allowed to develop EGID during follow-up (in which case they were censored and regarded as EGID cases).

#### Sibling comparators

To decrease confounding due to familial and early environmental factors we identified a secondary control cohort consisting of siblings (*n* = 2785) to EGID patients (1513 of these had a sibling). Siblings were retrieved from the TPR and were not allowed to have a diagnosis of EGID. Data on siblings are available on all individuals born after 1932 and who were registered residents of Sweden after 1961.

#### Outcome measure

We examined overall mortality, cause-specific mortality and cancer (any cancer and specific cancers). For *overall mortality*, we obtained data on death dates through the TPR (available data until 31 Dec. 2017). This register covers virtually 100% of deaths [16]. *Cause-specific mortality* data were obtained from the Swedish Cause of Death Register [17]. This validated register began in 1952 and has a high coverage. Deaths were divided into cardiovascular, cancer and other deaths.

*Cancer* data were retrieved from the Swedish Cancer Register. Reporting of cancer data is compulsory in Sweden, and it has been estimated that this register has a coverage of 96.3% [18]. The Cancer Register uses ICD7-codes to specify cancer diagnoses. Cancers were divided into GI cancer, skin cancer (melanoma and non-melanoma), lung cancer, breast cancer and hematologic cancers (Supplemental Table 4). Subgroup analysis was completed by site of cancer and distribution of eosinophilia. GI cancers were divided into luminal cancers (esophageal, gastric, small bowel, and colorectal) and pancreaticobiliary (pancreas, liver, and gallbladder) by ICD7 codes (150–154 and 155–158, respectively).

### Other covariates

Education level was divided into three categories: compulsory (≤ 9 years), upper secondary (10–12 years), and college or university (≥ 13 years), and obtained from the Longitudinal Integrated Database for Health Insurance and Labour Market Studies (LISA) [19]. To examine if drug treatment in EGID had any impact on mortality or cancer development, we calculated separate aHRs for death and cancer in patients with and without steroids (ATC code of Budesonide/prednisolone (A07EA) or oral steroids (H02AB)) and proton-pump inhibitors (PPIs, ATC code: A02BC). Medication data were retrieved from the Swedish Prescribed Drug Register [20] and these analyses were hence restricted to incident EGID diagnosed from January 1, 2006 or later, when the register started. Medication use was defined as having a prescription between 7 days before and 30 days after biopsy date.

### Statistics

In the main analysis, we adjusted for age at EGID diagnosis (first/initial biopsy), sex, county of residence, and calendar year (“model 1”). In our fully adjusted model (“model 2”) we added the following covariates to our model: education (from the LISA database), eczema, asthma, allergy (all three from the Patient Register), and EoE (from the ESPRESSO cohort) (Supplemental Table 5).

Study follow-up began on date of EGID diagnosis (or matching date in reference individuals), and ended with death, emigration, or end of follow-up on December 31, 2017, whichever came first. For analyses on cause-specific death and cancer incidence, end of follow-up was December 31, 2016). Reference individuals were also censored if they developed EGID during follow-up and then moved to the EGID group. We used Cox proportional hazard modeling to calculate hazard ratios (HRs) and 95% confidence interval (95%CI) for overall and cause-specific mortality. Absolute risks (deaths per 1000 person-years of follow-up) were calculated. Finally, we carried out stratified analyses according to years of follow-up (divided into three groups), age at first EGID diagnosis (< 18, 18 to < 50, ≥ 50 years), sex, and education level (Table 1). Missing data were handled as separate categories.

**Table 1:** EGID patients and general population reference individuals

	EGID	Population reference individuals
	*n* [%]	*n* [%]
Total	2429 [100.00]	11,719 [100.00]
Male	1079 [44.42]	5182 [44.22]
Female	1350 [55.58]	6537 [55.78]
*Age at Start of Follow-up (years)*		
Mean [SD]	44.10 [22.65]	43.44 [22.48]
Median [IQR]	46.00 [26.00–62.00]	45.00 [25.00–61.00]
< 18	272 [11.20]	1327 [11.32]
18—< 50	996 [41.00]	4914 [41.93]
≥ 50	1161 [47.80]	5478 [46.74]
*Follow-up (years)*		
Mean [SD]	9.45 [6.07]	9.65 [6.10]
Median [IQR]	8.22 [4.25–14.17]	8.35 [4.37–14.46]
< 1	48 [1.98]	170 [1.45]
1 < 5	707 [29.11]	3361 [28.68]
≥ 5	1674 [68.92]	8188 [69.87]
*Calendar Year of Start of Follow -up*		
1990—2005	966 [39.77]	4679 [39.93]
2006—2013	1043 [42.94]	5047 [43.07]
2014—2017	420 [17.29]	1993 [17.01]
*Reasons for censoring*		
Emigration	26 [1.07]	196 [1.67]
End of follow-up (31 Dec. 2017)	2089 [86.00]	10,283 [87.75]
Diagnosed with EGID	0 [0.00]	0 [0.00]
Death	314 [12.93]	1240 [10.58]
–Cardiovascular diseases	98 [4.03]	439 [3.75]
–Cancer	87 [3.58]	313 [2.67]
–Other	129 [5.31]	488 [4.16]
*Education*		
Compulsory school (≤ 9 years)	613 [25.24]	2654 [22.65]
Upper secondary school (10–12 years)	879 [36.19]	4376 [37.34]
College or university (≥ 13 years)	567 [23.34]	2894 [24.69]
NA	370 [15.23]	1795 [15.32]

*EGID* eosinophilic gastrointestinal disorders distal to the esophagus

*NA* no data available

In a secondary comparison, the rate of mortality in EGID patients was compared with their siblings. Sibling analyses were stratified as per family (one stratum per family). The advantage of a sibling approach is that it automatically references individuals for covariates that are shared in the family (family situation, genetics, etc.).

Corticosteroid exposure and proton pump inhibitor (PPI) exposure were assessed in both EGID subjects and in reference individuals. Influence on mortality and cancer risk were calculated. These analyses were limited to individuals diagnosed with EGID from January 1, 2006 to allow for six months of exposure since the Swedish Prescribed Drug Register only started in July 2005 [20]. Drug analyses were adjusted for all variables in model 2. (Supplemental Table 5).

To evaluate for any potential confounding from smoking, alcohol use, obesity, or inflammatory bowel disease, sensitivity analyses were completed. ICD codes for obesity, alcohol-related disorders, and chronic obstructive pulmonary disease (COPD) (as a proxy for smoking) were used and hazard ratios for death and cancer were calculated adjusted for these variables [21, 22]. Similarly, we excluded any subjects with inflammatory bowel disease at baseline, defined according to recent study (≥ 1 colorectal biopsy with inflammation plus ≥ 1 ICD code) [23]. This latter definition has a positive predictive value of 95% [24]. Again, hazard ratios for death and cancer were calculated (Supplemental Table 6).

Finally, a secondary analysis was completed in those with eosinophilic colitis to determine the number that had concurrent or subsequent (7 days prior or ever after diagnosis of eosinophilic colitis) diagnosis of eosinophilic gastritis or gastroenteritis, no evidence of eosinophilic gastritis or gastroenteritis on biopsy, or no biopsies to have the opportunity to be diagnosed with eosinophilic gastritis/gastroenteritis.

Statistics were carried out using R statistical software (version 3.5.2, R Foundation for Statistical Computing, Vienna, Austria) and the survival package (version 2.43, Therneau, T (2015), https://CRAN.R-project.org/package=survival). Statistical significance was set to *p* < 0.05. CIs were computed by inversion of the likelihood ratio test statistic.

### Ethics

The study was approved by the Stockholm Ethics Authority. Informed consent was waived given that the study was strictly register-based [25].

## Results

### Background data of EGID patients and reference individuals

In total, 2429 subjects (56% female) were included for analysis and 11,719 matched reference individuals. Mean age was 44.1 years with 272 (11.2%) being children (< 18 years) at the time of diagnosis. Average follow up was 9.4 years with 1674 (68.9%) having > 5 years of follow up. Education levels were assessed and reason for ending follow up were described (Table 1).

Based on initial diagnostic biopsy, eosinophilic gastritis was seen in 292 subjects, eosinophilic gastroenteritis (small bowel disease) in 694, and eosinophilic colitis in 1756 (Table 2). When excluding eosinophilic disease of the esophagus, the prevalence of EGIDs in the Swedish population was 1/4763 or 21/100,000 (2017 Swedish population was 9.95 million; estimate based on 2089 EGID patients alive in 2017 and with no record of emigration during follow-up) (Table 1). EGID based on location of disease and year of diagnosis by 5-year strata were reported (Supplemental Table 7).

**Table 2:** Overview table with mortality rates and hazard ratios per cause of death outcome and exposure group

Outcome	Reference Individuals	All EGID	Eosinophilic Gastritis	Eosinophilic Gastroenteritis (small intestine)	Eosinophilic Colitis
*All-cause Death*					
N	11,719*	2429*	292*	694*	1756*
Events	1240	314	54	94	196
Person-years (*1000)	113.09	22.96	2.37	7.11	16.9
IR (%) [95% CI]	10.96 [10.36–11.59]	13.68 [12.21–15.28]	22.83 [17.15–29.79]	13.22 [10.68–16.18]	11.60 [10.03–13.34]
IR diff. [95% CI]	0 (ref.)	2.71 [1.08–4.34]	11.82 [5.71–17.93]	2.26 [-0.49–5]	0.63 [-1.1–2.37]
Model 1 aHR [95% CI]	1 (ref.)	1.19 [1.05–1.35]	1.81 [1.33–2.47]	1.53 [1.21–1.92]	1.00 [0.86–1.17]
Model 2 aHR [95% CI]	1 (ref.)	1.17 [1.04–1.33]	1.81 [1.32–2.48]	1.50 [1.18–1.89]	0.99 [0.85–1.16]
*Cardiovascular death*					
N	11,686	2422	290	691	1753
Events	439	98	22	26	57
Person-years (*1000)	113.06	22.95	2.36	7.11	16.89
IR (%) [95% CI]	3.88 [3.53–4.26]	4.27 [3.47–5.20]	9.31 [5.83–14.09]	3.66 [2.39–5.36]	3.37 [2.56–4.37]
IR diff. [95% CI]	0 (ref.)	0.39 [-0.53–1.31]	5.44 [1.53–9.35]	-0.23 [-1.68–1.23]	-0.51 [-1.46–0.44]
Model 1 aHR [95% CI]	1 (ref.)	1.03 [0.83–1.29]	1.88 [1.15–3.08]	1.23 [0.80–1.89]	0.82 [0.62–1.09]
Model 2 aHR [95% CI]	1 (ref.)	1.02 [0.82–1.28]	1.94 [1.18–3.20]	1.22 [0.79–1.88]	0.82 [0.62–1.09]
*Cancer death*					
*N*	11,686	2422	290	691	1753
Events	313	87	17	27	52
Person-years (*1000)	113.06	22.95	2.36	7.11	16.89
IR (%) [95% CI]	2.77 [2.47–3.09]	3.79 [3.04–4.68]	7.19 [4.19–11.52]	3.80 [2.50–5.53]	3.08 [2.30–4.04]
IR diff. [95% CI]	0 (ref.)	1.02 [0.17–1.88]	4.43 [1–7.87]	1.03 [-0.44–2.49]	0.31 [-0.58–1.2]
Model 1 aHR [95% CI]	1 (ref.)	1.33 [1.05–1.69]	2.76 [1.55–4.93]	1.77 [1.14–2.75]	1.05 [0.77–1.42]
Model 2 aHR [95% CI]	1 (ref.)	1.33 [1.05–1.69]	2.80 [1.56–5.02]	1.73 [1.11–2.70]	1.05 [0.78–1.42]
*Cancer death*					
N	11,686	2422	290	691	1753
Events	488	129	15	41	87
Person-years (*1000)	113.06	22.95	2.36	7.11	16.89
IR (%) [95% CI]	4.32 [3.94–4.72]	5.62 [4.69–6.68]	6.35 [3.55–10.47]	5.77 [4.14–7.82]	5.15 [4.12–6.35]
IR diff. [95% CI]	0 (ref.)	1.3 [0.26–2.35]	2.04 [-1.2–5.28]	1.45 [-0.36–3.26]	0.83 [-0.31–1.98]
Model 1 aHR [95% CI]	1 (ref.)	1.24 [1.02–1.51]	1.21 [0.69–2.14]	1.61 [1.13–2.29]	1.13 [0.90–1.44]
Model 2 aHR [95% CI]	1 (ref.)	1.20 [0.98–1.46]	1.17 [0.65–2.10]	1.55 [1.08–2.22]	1.10 [0.86–1.39]

*EGID* eosinophilic gastrointestinal disorders distal to the esophagus, *IR* incidence rate, *aHR* adjusted hazard ratios

Model 1 adjusts for age at EGID diagnosis (first biopsy), sex, county of residence, and calendar year. Model 2 adds adjustments for education, eczema, allergy, asthma, and eosinophilic esophagitis

^*^The number of study participants was slightly higher in the overall mortality than in the cause-specific mortality analyses. The follow-up of the cause-specific mortality analyses ended on 31 December 2016 and hence did not include EGID patients diagnosed in 2017 and their controls

The study evaluated the number of patients with concurrent diagnosis of EoE with a more distal EGID. In patients with any EGID distal to the esophagus, EoE was seen in 73 (3%). For those with eosinophilic gastritis and gastroenteritis, 49 (16.8%) and 34 (4.9%) had concurrent diagnosis of EoE, respectively. EoE was seen less frequently in those with eosinophilic colitis (23 patients (1.3%)).

Given the high frequency of eosinophilic colitis seen, secondary analysis was done to determine those that had the opportunity for concurrent or subsequent diagnosis of either eosinophilic gastritis or eosinophilic gastroenteritis. Of the 1756 with colonic disease, 239 (14%) had concurrent or subsequent diagnosis of eosinophilic gastritis or gastroenteritis, 726 (41%) had concurrent or subsequent EGD without diagnosis of more proximal disease, and 791 (45%) did not have concurrent or subsequent EGD to diagnose eosinophilic gastritis or gastroenteritis.

### Overall mortality

**Fig. 1:**
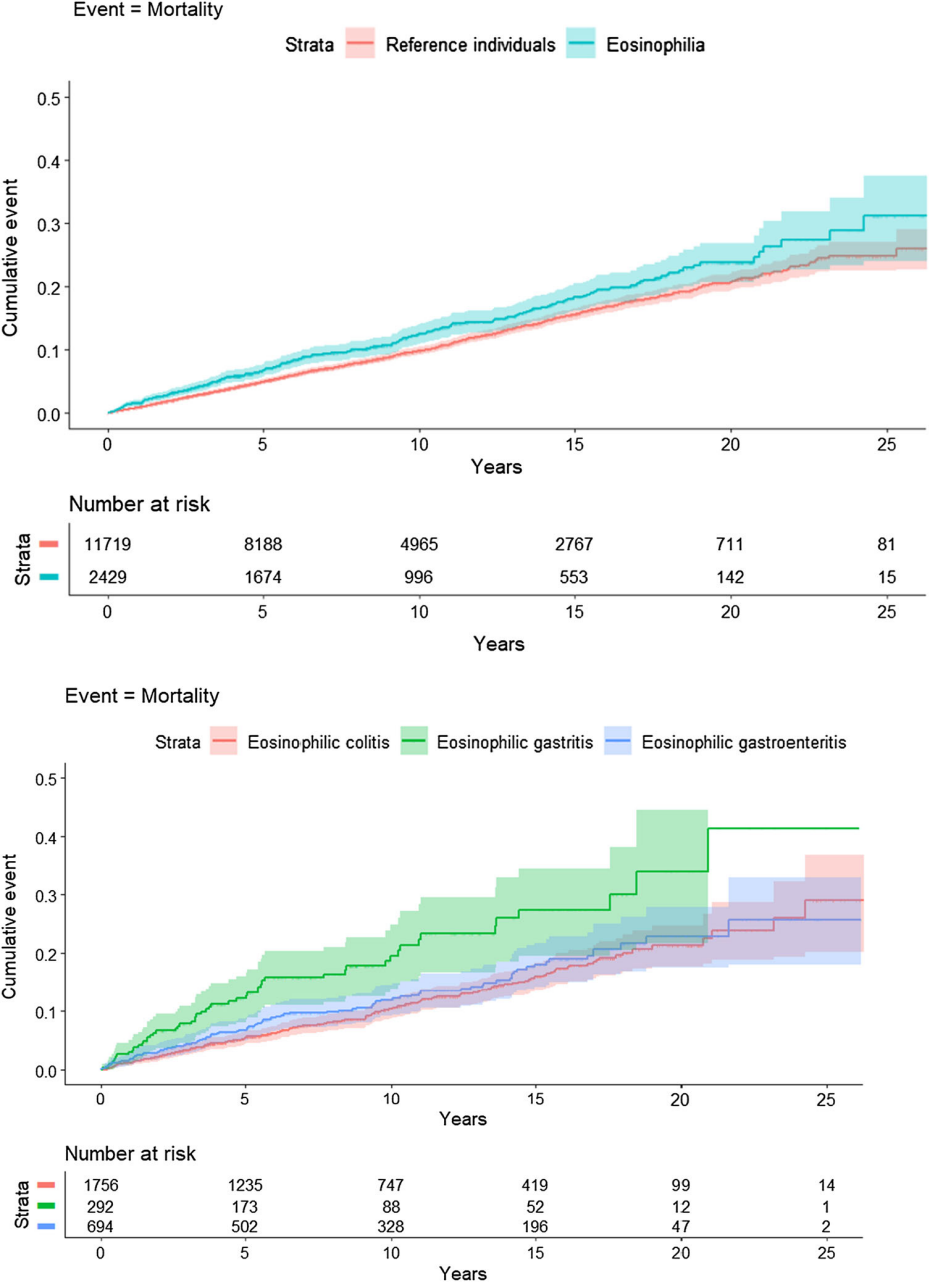
Kaplan–Meier plots describing mortality in EGID patients compared to controls and based on distribution of eosinophilia. Figure 1 (upper) illustrates mortality over time in patients with eosinophilic gastrointestinal disorders (EGID) distal to the esophagus compared to reference individuals showing separation in mortality over time. Figure 1 (lower) demonstrates mortality over time in EGID distal to the esophagus stratified by location of eosinophilia

With follow up of approximately 23,000 person-years, there were 314 deaths in the cohort with EGID. Incidence rate for death was 13.68/1000 person-years in the EGID cohort and 10.96/1000 person-years in the reference cohort, with an absolute increased incidence of 2.71/1000 person-years. Adjusting for potential confounders, the data showed a 17% increased hazard of death in the cohort with EGID compared to reference individuals (aHR = 1.17; 95%CI = 1.04–1.33). The risk was driven by foregut disease which included an 81% increased mortality in subjects with eosinophilic gastritis (aHR = 1.81; 95%CI = 1.32–2.48) and a 50% increased mortality in eosinophilic gastroenteritis (aHR = 1.50; 95%CI = 1.18–1.89). No increase in mortality was seen in those with eosinophilic colitis (aHR = 0.99; 95%CI = 0.85–1.16) (Table 2, Fig. 1). Sensitivity analysis adjusting for COPD (proxy for smoking), alcohol-related disorders, and obesity had marginal effect on mortality (HR 1.14; 95%CI = 1.01–1.30). Similarly, excluding individuals with prior inflammatory bowel disease had limited effect on mortality (HR 1.20; 95%CI = 1.06–1.37).

### Cause-specific mortality

In the 314 subjects who died during follow up, cancer mortality was increased 33% compared to reference individuals (aHR = 1.33; 95%CI = 1.05–1.69). Overall cardiovascular death and death from other causes was not increased when compared to reference individuals (aHR = 1.02; 95%CI = 0.82–1.28 and aHR = 1.20; 95%CI = 0.98–1.46, respectively). In subgroup analyses, there was an increased risk of cardiovascular death in those with eosinophilic gastritis (aHR = 1.94; 95%CI = 1.18–3.20) and death from other causes in those with eosinophilic gastroenteritis (aHR = 1.55; 95%CI = 1.08–2.22), understanding that these subgroup data could be confounded by small numbers and multiple comparisons (Table 2).

Cancer related death was increased in the EGID cohort compared to reference individuals (aHR = 1.33; 95%CI = 1.05–1.69). Foregut disease again seemed to drive this increase with a 180% increase in those with eosinophilic gastritis (aHR = 2.80; 95%CI = 1.56–5.02) and 73% increase in eosinophilic gastroenteritis (aHR = 1.73; 95%CI = 1.11–2.70). No increased cancer mortality was seen in those with eosinophilic colitis (Table 2).

### Cancer incidence

During follow up, 175 subjects developed incident cancer after EGID diagnosis, which was not significantly increased compared to reference individuals (aHR = 1.14; 95%CI = 0.96–1.35). There was limited power to detect differences in cancer subtypes but trends toward increased risk were seen in hematologic malignancies (aHR = 1.78; 95%CI = 1.01–3.12) and GI malignancies (aHR = 1.43; 95%CI = 0.95–2.15) (Table 3), again driven by eosinophilic gastritis. Sensitivity analysis controlling for COPD (proxy for smoking), alcohol-related disorders, and obesity did not impact or have association with cancer risk (HR 1.11; 95%CI = 0.94–1.31). Similarly, excluding individuals with prior inflammatory bowel disease did not change result on cancer risk (HR 1.12; 95% CI = 0.94–1.35).

**Table 3:** Overview table with incidence rates and hazard ratios per cancer outcome and exposure group

Outcome	Reference Individuals	All EGID	Eosinophilic Gastritis	Eosinophilic Gastroenteritis (small intestine)	Eosinophilic Colitis
*All-cause Death*					
N	11,719*	2429*	292*	694*	1756*
Events	1240	314	54	94	196
IR (%) [95% CI]	10.96 [10.36–11.59]	13.68 [12.21–15.28]	22.83 [17.15–29.79]	13.22 [10.68–16.18]	11.60 [10.03–13.34]
IR diff. [95% CI]	0 (ref.)	2.71 [1.08–4.34]	11.82 [5.71–17.93]	2.26 [-0.49–5]	0.63 [-1.1–2.37]
Model 1 aHR [95% CI]	1 (ref.)	1.19 [1.05–1.35]	1.81 [1.33–2.47]	1.53 [1.21–1.92]	1.00 [0.86–1.17]
Model 2 aHR [95% CI]	1 (ref.)	1.17 [1.04–1.33]	1.81 [1.32–2.48]	1.50 [1.18–1.89]	0.99 [0.85–1.16]
*Cardiovascular Death*					
N	11,686	2422	290	691	1753
Events	439	98	22	26	57
Person-years (*1000)	113.06	22.95	2.36	7.11	16.89
IR (%) [95% CI]	3.88 [3.53–4.26]	4.27 [3.47–5.20]	9.31 [5.83–14.09]	3.66 [2.39–5.36]	3.37 [2.56–4.37]
IR diff. [95% CI]	0 (ref.)	0.39 [-0.53–1.31]	5.44 [1.53–9.35]	-0.23 [-1.68–1.23]	-0.51 [-1.46–0.44]
Model 1 aHR [95% CI]	1 (ref.)	1.03 [0.83–1.29]	1.88 [1.15–3.08]	1.23 [0.80–1.89]	0.82 [0.62–1.09]
Model 2 aHR [95% CI]	1 (ref.)	1.02 [0.82–1.28]	1.94 [1.18–3.20]	1.22 [0.79–1.88]	0.82 [0.62–1.09]
*Cancer Death*					
*N*	11,686	2422	290	691	1753
Events	313	87	17	27	52
Person-years (*1000)	113.06	22.95	2.36	7.11	16.89
IR (%) [95% CI]	2.77 [2.47–3.09]	3.79 [3.04–4.68]	7.19 [4.19–11.52]	3.80 [2.50–5.53]	3.08 [2.30–4.04]
IR diff. [95% CI]	0 (ref.)	1.02 [0.17–1.88]	4.43 [1–7.87]	1.03 [-0.44–2.49]	0.31 [-0.58–1.2]
Model 1 aHR [95% CI]	1 (ref.)	1.33 [1.05–1.69]	2.76 [1.55–4.93]	1.77 [1.14–2.75]	1.05 [0.77–1.42]
Model 2 aHR [95% CI]	1 (ref.)	1.33 [1.05–1.69]	2.80 [1.56–5.02]	1.73 [1.11–2.70]	1.05 [0.78–1.42]
*Other Death*					
*N*	11,686	2422	290	691	1753
Events	488	129	15	41	87
Person-years (*1000)	113.06	22.95	2.36	7.11	16.89
IR (%) [95% CI]	4.32 [3.94–4.72]	5.62 [4.69–6.68]	6.35 [3.55–10.47]	5.77 [4.14–7.82]	5.15 [4.12–6.35]
IR diff. [95% CI]	0 (ref.)	1.3 [0.26–2.35]	2.04 [-1.2–5.28]	1.45 [-0.36–3.26]	0.83 [-0.31–1.98]
Model 1 aHR [95% CI]	1 (ref.)	1.24 [1.02–1.51]	1.21 [0.69–2.14]	1.61 [1.13–2.29]	1.13 [0.90–1.44]
Model 2 aHR [95% CI]	1 (ref.)	1.20 [0.98–1.46]	1.17 [0.65–2.10]	1.55 [1.08–2.22]	1.10 [0.86–1.39]

*EGID* eosinophilic gastrointestinal disorders distal to the esophagus, *IR* incidence rate, *aHR* adjusted hazard ratios

Model 1 adjusts for age at EGID diagnosis (first biopsy), sex, county of residence, and calendar year. Model 2 adds adjustments for education, eczema, allergy, asthma, and eosinophilic esophagitis

When evaluating cancer association with the anatomic site of eosinophilic involvement, there was no association between EG and gastric cancer (no gastric cancers in those with EG, aHR 95% CI not calculated), EGE and small bowel cancer (aHR 5.38; 95%CI = 0.33–88.02), or EC and colorectal cancer (aHR 1.04; 95%CI = 0.55–1.97), but statistical power was limited.

Finally, two subgroup analyses were completed evaluating association between EGIDs distal to the esophagus and association with luminal and pancreaticobiliary cancers. There was no association between all EGIDs distal to the esophagus and luminal cancer (aHR 1.28; 95%CI = 0.82–1.98), but an association was suggested between EG and luminal cancer (aHR 3.77; 95%CI = 1.33–10.65). There was a suggested association between EGIDs distal to the esophagus and pancreaticobiliary cancers (aHR 2.51; 95%CI = 1.23–5.11), driven by eosinophilic colitis (aHR 2.72; 95%CI = 1.17–6.32) (Supplemental Table 8). Caution is urged in interpreting these subgroup analyses given the small numbers of cancer events.

### Additional analyses

Sibling analysis was completed to assess for genetic and shared environmental contributors to mortality. We included 1513 of the EGID subjects and 2785 siblings in the analysis (Table 4). Overall mortality hazard mirrored the main analysis (aHR = 1.26; 95%CI = 0.93–1.70), though this did not meet statistical significance (Table 5). Compared to siblings, EGID patients were at no increased risk of incident cancer (aHR = 1.27; 95%CI = 0.94–1.70) (Supplemental Table 9).

**Table 4:** Summary statistics for EGID patients and their siblings

	EGID	Siblings
	*n* [%]	*n* [%]
Total	1513 [100.00]	2785 [100.00]
Male	696 [46.00]	1395 [50.09]
Female	817 [54.00]	1390 [49.91]
*Age at Start of Follow-up (years)*		
Mean [SD]	38.96 [20.06]	40.49 [19.95]
Median [IQR]	40.00 [22.00–55.00]	42.00 [24.00–57.00]
< 18	194 [12.82]	270 [9.69]
18—< 50	723 [47.79]	1363 [48.94]
≥ 50	596 [39.39]	1152 [41.36]
*Follow-up (years)*		
Mean [SD]	9.87 [6.16]	10.06 [6.23]
Median [IQR]	8.59 [4.51–14.88]	8.63 [4.62–15.05]
< 1	19 [1.26]	23 [0.83]
1 < 5	415 [27.43]	756 [27.15]
≥ 5	1079 [71.32]	2006 [72.03]
*Calendar Year of Start of Follow-up*		
1990—2005	582 [38.47]	1109 [39.82]
2006—2013	661 [43.69]	1195 [42.91]
2014—2017	270 [17.85]	481 [17.27]
*Reason for Censoring*		
Emigration	12 [0.79]	34 [1.22]
End of follow-up (31 Dec. 2017)	1408 [93.06]	2598 [93.29]
Diagnosed with EGID	0 [0.00]	3 [0.11]
Death	93 [6.15]	150 [5.39]
–Cardiovascular diseases	19 [1.26]	32 [1.15]
–Cancer	40 [2.64]	64 [2.30]
–Other	34 [2.25]	54 [1.94]
*Education*		
Compulsory school (≤ 9 years)	322 [21.28]	622 [22.33]
Upper secondary school (10–12 years)	567 [37.48]	1124 [40.36]
College or university (≥ 13 years)	383 [25.31]	620 [22.26]
NA	241 [15.93]	419 [15.04]

*EGID* eosinophilic gastrointestinal disorders distal to the esophagus

*NA* no data available

**Table 5:** All cause morality and cause specific mortality in EGID patients compared to siblings

Outcome	Siblings	EGID
*All-cause Death*		
*N*	2785*	1513*
Events	150	93
Person-years (*1000)	28.01	14.94
IR (%) [95% CI]	5.36 [4.53–6.28]	6.23 [5.03–7.63]
IR diff. [95% CI]	0 (ref.)	0.87 [-0.66–2.4]
Model 1 aHR [95% CI]	1 (ref.)	1.31 [0.98–1.75]
Model 2 aHR [95% CI]	1 (ref.)	1.26 [0.93–1.70]
*Cardiovascular Death*		
*N*	2778	1509
Events	32	19
Person-years (*1000)	28	14.93
IR (%) [95% CI]	1.14 [0.78–1.61]	1.27 [0.77–1.99]
IR diff. [95% CI]	0 (ref.)	0.13 [-0.57–0.83]
Model 1 aHR [95% CI]	1 (ref.)	0.87 [0.40–1.89]
Model 2 aHR [95% CI]	1 (ref.)	0.94 [0.42–2.09]
*Cancer Death*		
*N*	2778	1509
Events	64	40
Person-years (*1000)	28	14.93
IR (%) [95% CI]	2.29 [1.76–2.92]	2.68 [1.91–3.65]
IR diff. [95% CI]	0 (ref.)	0.39 [-0.61–1.39]
Model 1 aHR [95% CI]	1 (ref.)	1.43 [0.92–2.22]
Model 2 aHR [95% CI]	1 (ref.)	1.37 [0.87–2.18]
*Other Death*		
*N*	2778	1509
Events	54	34
Person-years (*1000)	28	14.93
IR (%) [95% CI]	1.93 [1.45–2.52]	2.28 [1.58–3.18]
IR diff. [95% CI]	0 (ref.)	0.35 [-0.57–1.27]
Model 1 aHR [95% CI]	1 (ref.)	1.39 [0.87–2.22]
Model 2 aHR [95% CI]	1 (ref.)	1.32 [0.80–2.19]

*EGID* eosinophilic gastrointestinal disorders distal to the esophagus, *IR* incidence rate, *aHR* adjusted hazard ratios

Model 1 adjusts for age at EGID diagnosis (first biopsy), sex, county of residence, and calendar year. Model 2 adds adjustments for education, eczema, allergy, asthma, and eosinophilic esophagitis

^*^The number of study participants was slightly higher in the overall mortality than in the cause-specific mortality analyses. The follow-up of the cause-specific mortality analyses ended on 31 December 2016 and hence did not include EGID patients diagnosed in 2017 and their controls

Corticosteroid and PPI exposure were assessed in EGID subjects and controls. Influence on mortality and cancer risk were calculated. In the mortality analysis, 102/1464 EGID patients had steroid exposure and 93/1298 in the cancer analysis. Only 99/1464 patients with EGID had PPI exposure in the mortality analysis and 87/1298 in the cancer analysis (Supplemental Table 10).

When adjusting for age, sex, county, year, education, asthma, eczema, allergy, and EoE, compared to matched controls, mortality in EGID patients with corticosteroids (aHR = 1.39; 95%CI = 0.61–3.15) and without corticosteroids (1.11; 95%CI = 0.90–1.38) were similar (*P* = 0.608). Corticosteroids did not impact on the risk of cancer in EGID patients (compared to the general population: aHR = 1.33; 95%CI = 0.52–3.42 vs 1.08; 95%CI = 0.81–1.43 respectively; *P* = 0.678). In contrast, EGID individuals with a record of PPI had a higher mortality than the general population (aHR = 2.36; 95%CI = 1.35–4.11), but this was not seen for EGID patients without PPIs (aHR = 1.05; 95%CI = 0.84–1.31) (*P* = 0.008). Similarly, PPI use was associated with increased risk of incident cancer in EGID (aHR = 3.29; 95%CI = 1.13–9.55 for users) compared to EGID patients without PPI (aHR = 1.05; 95%CI = 0.79–1.40) (*P* = 0.043) (Supplemental Table 10). We urge caution when interpreting data related to drug exposure since numbers were small.

## Discussion

We identified more than 2400 individuals with EGID. Patients with EGID were at a small, but statistically significantly increased risk of death (HR = 1.17; 95%CI = 1.04–1.33). The excess risk was limited to patients with eosinophilic gastritis and gastroenteritis, and not seen in patients with eosinophilic colitis (HR = 0.99). The highest mortality HRs were seen for death from cancer, and this excess risk was again limited to eosinophilic gastritis and gastroenteritis.

Eosinophilic disease of the foregut (eosinophilic gastritis and gastroenteritis) appear to be the driver for increased mortality and incident cancers of the GI tract and hematologic cancers. These data are somewhat diluted in the greater EGID cohort by the large number of patients with colonic disease, which does not seem to portend increased mortality or cancer risk. We failed to demonstrate an increased risk of incident cancer in the overall cohort (HR = 1.14; 95%CI = 0.96–1.35), likely due to limited power and effect from large group with colonic disease. Overall, the findings were confirmed in sibling analyses.

Our data suggest a prevalence at nearly 1/4800 (or 21/100,000), comparable to the higher end of previously reported data [3, 6, 8]. Furthermore, this study suggests a slightly higher prevalence in females at 56%, more in line with Spergel, et al*.* and differing from earlier reports suggesting slight male predominance [3, 7] To our knowledge, this is the first study describing mortality and cancer risk in EGIDs distal to the esophagus and the first true population-based study. Studies have not linked EoE with increased esophageal cancer risk [26].

Eosinophilic infiltration has been described as protective in some malignancies and detrimental in others [27]. This may be related to differing tissue- and tumor-specific profiles of recruited cytokines that have both protective effects and tumor stimulatory effects depending on the context [28]. The underlying mechanisms may be related to chronic inflammation leading to cancer, as described in other conditions such as inflammatory bowel disease [29–31], to direct effects of tumor infiltrating eosinophils secreting growth factors and immunosuppressive cytokines, or to an intermediate factor such as H. pylori infection. It is also possible that mucosal eosinophilia associated with cancer drives more symptoms and leads individuals to seek evaluation.

We show an increase in mortality among EGID patients compared to the general population. This contrasts the lack of excess mortality in the recent study of 1625 Swedish EoE patients (HR = 0.97; 95%CI = 0.67–1.40)[14]. We note the confidence intervals of the studies overlap.

Increased mortality in EGID was driven by cancer, despite cancer incidence not being increased. This may be due to statistical power, and we cannot rule out an increased risk of incident cancers in those with eosinophilic gastritis or gastroenteritis. In a recent paper evaluating mortality in EoE, there was no association with cancer mortality (HR = 1.05; 95%CI = 0.31–3.61), including GI cancer mortality[14]. This may represent a true lack of association. Alternatively, we speculate that corticosteroid exposure could mask a cancer diagnosis until later stages, but medication exposure analysis for corticosteroids does not seem to influence incident cancers. Another hypothesis is that patients with EGIDs often live with many symptoms attributed to their EGID without efficacious therapies available. This could lead to later diagnoses of cancer, with patients and providers attributing symptoms to their EGID (anchoring bias). Finally, enteric eosinophilia could represent a marker of poorer prognosis and lead to increased cancer mortality without increased cancer incidence. These hypotheses warrant further study.

Several subgroup analyses were assessed looking at association with cancer in the organ involved with the EGID and with association between EGID and luminal versus pancreaticobiliary cancers. We did not demonstrate an association between the location of the EGID and cancer in the affected organ. Overall, luminal cancers were not associated with EGIDs distal to the esophagus, but there was some suggestion again of proximal disease increasing risk. Finally, there was a possible association with EGIDs and pancreaticobiliary cancers, predominantly associated with colonic eosinophilia. These data raise interesting questions, but given the very small numbers of cancers, should be interpreted cautiously.

This study has several strengths. Data was collected from all 28 of Sweden’s pathology departments. The nationwide approach minimizes the risk of surveillance bias, which occurs when patients originate from specialized centers and may inflate relative risks. We based our EGID definition on biopsy reports. We did not have the ethics approval to validate these with slide review, but patient chart reviews of EoE—a similar disorder—have yielded a positive predictive value of 89% [32]. We excluded individuals with disorders that mimic EGID, and we believe our definition has a high specificity. Although we cannot rule out a lower sensitivity, our cumulative prevalence data (21/100,000) is similar to what has been reported previously [3].

We used prospectively recorded data linked to Sweden’s healthcare registers, eliminating recall bias. Using the national Cause of Death and Cancer register, we had virtually no loss of follow-up (median follow-up > 8 years). Both registers have been validated and the Cancer register has a coverage of > 96% [18]. Our study included all ages. While children were few (*n* = 272), this constitutes the first attempt to calculate absolute and relative risks for death and cancer in children with EGID distal to the esophagus. We had data on 2429 individuals and the large numbers allowed for a number of sub-analyses and to evaluate cancer and mortality according to strata. We were able to examine different types of EGID, noting different risks in gastric and small intestinal EGID, compared to colorectal eosinophilia. We had data on many potential confounders, including education as a proxy for socioeconomic status, comorbidities including asthma and other eosinophilic disorders, and sensitivity analyses for medication use. Further sensitivity analyses considering smoking, obesity, alcohol, and inflammatory bowel disease were completed. All adjustments had only a marginal effect on the hazard estimates. Access to sibling data allowed us to exclude the influence of intrafamilial confounding.

This paper has several limitations. The dataset is likely restricted to patients with symptomatic disease since Sweden has no screening for EGID. While we adjusted for socioeconomic factors, we have no data on ethnicity. Sweden has seen increases in immigration but is still largely Caucasian. It may be difficult to extrapolate these data to countries with a different racial and ethnic demographics. Diagnostic criteria for EGID’s distal to the esophagus have not been well defined, especially during the earlier timeframes assessed in this study. However, the eosinophilia was significant enough to prompt the pathologist to code/remark in their report. Eosinophils are commonly seen in the GI tract, especially the colon, and the pathologist would deem abnormal to code for them in the pathology register. We had limited statistical power for specific causes of death and cancer. The Swedish pathology registers have a poor sensitivity for H. pylori infection, preventing evaluation of this potential confounder. Finally, we had no data on smoking status, but the HR of 0.74 for lung cancer suggests against smoking being overrepresented in EGID.

This study showed similar prevalence to prior reports [3], but a higher proportion of colonic disease. This could be due to erroneous inclusion due to histologic eosinophil counts not available in a register-based study. However, the pathologists coded in the pathology register an abnormality from what they would determine to be normal GI eosinophilia, and we excluded known secondary causes of eosinophilia. Another explanation is that many of those diagnosed with colonic disease did not have the opportunity to be diagnosed with proximal disease, with 45% of those never having pathology sampling from the stomach or duodenum at the time of diagnosis of eosinophilic colitis or anytime after. This may explain the similar prevalence to prior studies but higher proportion of colonic disease. Finally, this was a population-based study, differing from prior reports which were commercial insurance database (ages 0–64 with insurance) [6], data from 26 major medical centers [8], or nationwide survey-based requiring response [33].

Corticosteroid exposure did not appear to influence mortality or cancer, but PPI exposure did appear to be associated with mortality and incident cancers. These data need to be interpreted with caution given the small numbers, risk for selection bias, but does warrant further study.

In conclusion, this nationwide cohort study of some 2400 patients with EGID observed a small but significantly increased risk of death. This risk seems to be driven by cancer-related death.

## Supplementary Information

Below is the link to the electronic supplementary material.

Supplementary file1 (DOCX 36 kb)

## Data Availability

Other researchers can apply for our data through the participating Swedish pathology departments and the Swedish National Board of Health and Welfare. Transparency: The lead author affirms that this manuscript is an honest, accurate, and transparent account of the study being reported; that no important aspects of the study have been omitted; and that any discrepancies from the study as planned (and, if relevant, registered) have been explained.

## Abbreviations

aHR: Adjusted hazard ratio
CI: Confidence interval
COPD: Chronic obstructive pulmonary disease
EGID: Eosinophilic gastrointestinal disorders (distal to the esophagus)
EoE: Eosinophilic esophagitis
GI: Gastrointestinal
HR: Hazard ratio
IQR: Interquartile range
PPI: Proton-pump inhibitor
TPR: Total population register
