# Supplementary material for: Mortality and cancer in eosinophilic gastrointestinal disorders distal to the esophagus: nationwide cohort study 1990–2017
Source: J Gastroenterol. 2022 Jul 19;57(10):735–47. doi: 10.1007/s00535-022-01904-5 (PMC9522613; doi:10.1007/s00535-022-01904-5)
Supplement: Supplementary file 1 — Supplementary file1 (DOCX 36 kb) [file 535_2022_1904_MOESM1_ESM.docx]

**Supplemental Table 1: Inclusion diagnoses**

|  | Topography code | Morphology code |
| --- | --- | --- |
| Eosinophilia | T63-T68 (stomach🡪rectum) | M4715 |
| Eosinophilic gastritis (EG) | T63 | M4715 |
| Eosinophilic gastroenteritis (EGE) – first biopsy with EGE | T64-65 | M4715 |
| Eosinophilic colitis (EC) – first biopsy with EC | T67-T68 | M4715 |

**Supplemental Table 2: Exclusion diagnoses**

|  |  | ICD9 | ICD10 |
| --- | --- | --- | --- |
| Ever diagnosed | Hypereosinophilic syndrome/ Eosinophilic leukemia | - | D47.5 |
|  | Lung eosinophilia /Loeffler syndrome | 518D | J82 |
|  | Parasite infection | 084-086  120-136 | B50-83 |
|  | Eosinophilic granuloma/Churg-Strauss | 528X, 446 | M30.1 |
|  | Histiocytosis X | 202, 277W | C96.0, C96.5, C96.6 |
|  | Eosinophilic meningitis | 322B | B83.2 |
|  | Eosinophilic myocarditis | - | I42.3 |
|  | Hereditary Eosinophilia | 288D | D72.1B |
|  | DRESS | 995A | T78.4 |
| Last 12 months before biopsy | Adenocarcinoma (gastric, small intestinal, colorectal) | 152-154 | C16-C20 |

EGID, eosinophilic gastrointestinal disease distal to esophagus. DRESS, drug reaction with eosinophilia and systemic symptoms.

| **Supplemental Table 3: Patients excluded from both EGIDs and controls based on exclusion criteria** | | |
| --- | --- | --- |
|  | | |
|  | Exposed | Controls |
|  | | |
| Exclusion criteria |  |  |
| Eligible (N [%]) | 2864 [100.00] | 13989 [100.00] |
| Emigration prior to index date | 3 [0.10] | 108 [0.77] |
| Hyper Eosinophilic Syndrome | 1 [0.03] | 0 [0.00] |
| Eosinophilic Lung Disease | 3 [0.10] | 0 [0.00] |
| Parasite | 31 [1.08] | 19 [0.14] |
| Granuloma | 12 [0.42] | 3 [0.02] |
| Histiocytosis | 1 [0.03] | 2 [0.01] |
| Meningitis | 0 [0.00] | 0 [0.00] |
| Myocarditis | 0 [0.00] | 0 [0.00] |
| Hereditary | 5 [0.17] | 0 [0.00] |
| DRESS | 35 [1.22] | 46 [0.33] |
| Adenocarcinoma | 31 [1.08] | 7 [0.05] |
|  | | |

EGID, eosinophilic gastrointestinal disease distal to esophagus. DRESS, drug reaction with eosinophilia and systemic symptoms.

**Supplemental Table 4: Coding used for cancer analysis**

| Diagnosis of Incident Cancer | ICD 7 CODES |
| --- | --- |
| Any Incident Cancer | ICD 7: 140-208 |
| GI Cancer including liver cancer | 150-159 |
| Melanoma / non-melanoma skin Cancer | 190, 191 |
| Lung Cancer | 162 |
| Breast Cancer | 170 |
| Extra-hepatic Hematologic Cancers | 200-208 |

**Supplemental Table 5: Covariates used in fully adjusted model**

| Diagnosis | ICD9 | ICD10 | SnoMed |
| --- | --- | --- | --- |
| Eosinophilic Esophagitis |  |  | T62 with M4715 |
| Asthma | 493 | J45, J46 |  |
| Eczema | 691 | L20 |  |
| Allergy/Urticaria | 477, 708 | J30, H10, L50, D72.1A, Z91.0 |  |

ICD, international classification of disease

**Supplemental Table 6: Coding used for sensitivity analyses**

| **Diagnosis** | **ICD7** | **ICD8** | **ICD9** | **ICD10** |
| --- | --- | --- | --- | --- |
| **COPD (proxy for smoking)** |  | **490-496** | **490-496** | **J40-47** |
| **Alcohol-related Disorders*** | **280,00 281,00 307 322 581,10 583,10 960,20 960,29** | **261,00 262,00 291 303 571,00 571,01 980,00 980,01 980,99** | **291 303 357F 425F 535D 571A 571B 571C 571D 980A 980X 790D 977D V79B** | **F10 E244 G621 I426 K292 G312 G721 K701 K709 K703 K700 K702 K704 K852 K860 O354 T510 T519 R780 Y573 X65 Y90 Y91 Z502 Z714 Z721** |
| **Obesity** |  | **278 649.1** | **278 649B** | **E650-679** |
| **Inflammatory Bowel Disease** | **572,20 72,21 578,03 572,00 572,09** | **563,1 563,00 569,02 569,04** | **555-556** | **K50-51, K52.3** |

**COPD, chronic obstructive pulmonary disease.**

***Methodology for alcohol-related disorders from Bergman *et al.* [22].**

| **Supplemental Table 7: EGID diagnosis by location and year of diagnosis** | | | | |
| --- | --- | --- | --- | --- |
|  | | | | |
| YEARS  (Start of Follow-up) | EG  N [%] | EGE  N [%] | EC  N [%] |  |
| 1990 - 1995 | 10 [4.69] | 19 [4.91] | 58 [3.67] |  |
| 1996 - 2000 | 27 [12.68] | 69 [17.83] | 231 [14.61] |  |
| 2001 - 2005 | 27 [12.68] | 79 [20.41] | 364 [23.02] |  |
| 2006 - 2010 | 58 [27.23] | 97 [25.06] | 319 [20.18] |  |
| 2011 - 2015 | 79 [37.09] | 110 [28.42] | 584 [36.94] |  |
| 2016 - 2017 | 12 [5.63] | 13 [3.36] | 25 [1.58] |  |
|  | | | | |

EGID, eosinophilic gastrointestinal disease; EG, eosinophilic gastritis; EGE, eosinophilic gastroenteritis; EC, eosinophilic colitis

**Supplemental Table 8: Incident cancers by anatomic location and distribution of EGID**

| Outcome  All Gastrointestinal Cancers | N | Reference Individuals  10866 | All EGID  2181 | EG  250 | EGE  633 | EC  1581 |
| --- | --- | --- | --- | --- | --- | --- |
|  | Events | 135 | 39 | 7 | 9 | 26 |
|  | Person-years (*1000) | 106.09 | 21.09 | 2.06 | 6.67 | 15.55 |
|  | IR (%) [95% CI] | 1.27 [1.07-1.51] | 1.85 [1.32-2.53] | 3.39 [1.36-6.99] | 1.35 [0.62-2.56] | 1.67 [1.09-2.45] |
|  | IR diff. [95% CI] | 0 (ref.) | 0.58 [-0.04-1.2] | 2.13 [-0.4-4.65] | 0.08 [-0.83-0.98] | 0.4 [-0.28-1.08] |
|  | Model 1 aHR [95% CI] | 1 (ref.) | 1.52 [1.05-2.19] | 4.25 [1.59-11.34] | 1.26 [0.60-2.64] | 1.42 [0.91-2.21] |
|  | Model 2 aHR [95% CI] | 1 (ref.) | 1.51 [1.04-2.18] | 4.13 [1.55-11.03] | 1.14 [0.53-2.48] | 1.45 [0.92-2.26] |
|  |  |  |  |  |  |  |
| Luminal GI Cancers | N | 10866 | 2181 | 250 | 633 | 1581 |
|  | Events | 111 | 27 | 6 | 6 | 17 |
|  | Person-years (*1000) | 106.14 | 21.11 | 2.06 | 6.68 | 15.57 |
|  | IR (%) [95% CI] | 1.05 [0.86-1.26] | 1.28 [0.84-1.86] | 2.91 [1.07-6.33] | 0.90 [0.33-1.96] | 1.09 [0.64-1.75] |
|  | IR diff. [95% CI] | 0 (ref.) | 0.23 [-0.29-0.75] | 1.87 [-0.47-4.21] | -0.15 [-0.89-0.6] | 0.05 [-0.51-0.6] |
|  | Model 1 aHR [95% CI] | 1 (ref.) | 1.30 [0.84-1.99] | 3.82 [1.35-10.80] | 1.08 [0.45-2.64] | 1.13 [0.66-1.94] |
|  | Model 2 aHR [95% CI] | 1 (ref.) | 1.28 [0.82-1.98] | 3.77 [1.33-10.65] | 0.93 [0.36-2.43] | 1.16 [0.68-1.99] |
|  |  |  |  |  |  |  |
| Pancreaticobiliary GI Cancers | N | 10866 | 2181 | 250 | 633 | 1581 |
|  | Events | 24 | 12 | 1 | 3 | 9 |
|  | Person-years (*1000) | 106.54 | 21.18 | 2.09 | 6.7 | 15.59 |
|  | IR (%) [95% CI] | 0.23 [0.14-0.34] | 0.57 [0.29-0.99] | 0.48 [0.01-2.67] | 0.45 [0.09-1.31] | 0.58 [0.26-1.10] |
|  | IR diff. [95% CI] | 0 (ref.) | 0.34 [0.01-0.67] | 0.25 [-0.69-1.2] | 0.22 [-0.29-0.74] | 0.35 [-0.04-0.74] |
|  | Model 1 aHR [95% CI] | 1 (ref.) | 2.47 [1.21-5.03] | 10.67 [0.52-217.81] | 1.91 [0.49-7.42] | 2.70 [1.17-6.26] |
|  | Model 2 aHR [95% CI] | 1 (ref.) | 2.51 [1.23-5.11] | 12.08 [0.64-227.56] | 1.91 [0.49-7.44] | 2.72 [1.17-6.32] |
|  |  |  |  |  |  |  |

EGID, eosinophilic gastrointestinal disorders distal to the esophagus. EG, eosinophilic gastritis. EGE, eosinophilic gastroenteritis. EC, eosinophilic colitis. IR, incidence rate. aHR, adjusted hazard ratios.

Model 1 adjusts for age at EGID diagnosis (first biopsy), sex, county of residence, and calendar year.

Model 2 adds additional adjustments for education, eczema, allergy, asthma, and eosinophilic esophagitis.

| **Supplemental Table 9: Incident cancers in EGID patients compared to siblings** | | | |
| --- | --- | --- | --- |
|  | | | |
| Outcome |  | Siblings | EGID |
|  | | | |
| Any Cancer | N | 2598 | 1394 |
|  | Events | 172 | 94 |
|  | Person-years (*1000) | 25.82 | 13.62 |
|  | IR (%) [95% CI] | 6.66 [5.70-7.74] | 6.90 [5.58-8.45] |
|  | IR diff. [95% CI] | 0 (ref.) | 0.24 [-1.47-1.95] |
|  | Model 1 aHR [95% CI] | 1 (ref.) | 1.33 [1.00-1.78] |
|  | Model 2 aHR [95% CI] | 1 (ref.) | 1.27 [0.94-1.70] |
|  |  |  |  |
| Gastrointestinal | N | 2598 | 1394 |
|  | Events | 26 | 18 |
|  | Person-years (*1000) | 26.52 | 14 |
|  | IR (%) [95% CI] | 0.98 [0.64-1.44] | 1.29 [0.76-2.03] |
|  | IR diff. [95% CI] | 0 (ref.) | 0.31 [-0.4-1.01] |
|  | Model 1 aHR [95% CI] | 1 (ref.) | 2.74 [1.24-6.02] |
|  | Model 2 aHR [95% CI] | 1 (ref.) | 2.56 [1.13-5.78] |
|  |  |  |  |
| Skin | N | 2598 | 1394 |
|  | Events | 34 | 12 |
|  | Person-years (*1000) | 26.48 | 14.01 |
|  | IR (%) [95% CI] | 1.28 [0.89-1.79] | 0.86 [0.44-1.50] |
|  | IR diff. [95% CI] | 0 (ref.) | -0.43 [-1.08-0.22] |
|  | Model 1 aHR [95% CI] | 1 (ref.) | 0.49 [0.20-1.18] |
|  | Model 2 aHR [95% CI] | 1 (ref.) | 0.55 [0.23-1.36] |
|  |  |  |  |
| Lung | N | 2598 | 1394 |
|  | Events | 13 | 4 |
|  | Person-years (*1000) | 26.59 | 14.05 |
|  | IR (%) [95% CI] | 0.49 [0.26-0.84] | 0.28 [0.08-0.73] |
|  | IR diff. [95% CI] | 0 (ref.) | -0.2 [-0.59-0.18] |
|  | Model 1 aHR [95% CI] | 1 (ref.) | 0.82 [0.19-3.51] |
|  | Model 2 aHR [95% CI] | 1 (ref.) | 1.28 [0.21-7.92] |
|  |  |  |  |
| Breast | N | 2598 |  |
|  | Events | 17 |  |
|  | Person-years (*1000) | 26.5 |  |
|  | IR (%) [95% CI] | 0.64 [0.37-1.03] |  |
|  | IR diff. [95% CI] | 0 [-0.43-0.43] |  |
|  | Model 1 aHR [95% CI] | 17/26.50 |  |
|  | Model 2 aHR [95% CI] |  |  |
|  |  |  |  |
| Hematologic | N | 2598 |  |
|  | Events | 9 |  |
|  | Person-years (*1000) | 26.59 |  |
|  | IR (%) [95% CI] | 0.34 [0.15-0.64] |  |
|  | IR diff. [95% CI] | 0 [-0.31-0.31] |  |
|  | Model 1 aHR [95% CI] | 9/26.59 |  |
|  | Model 2 aHR [95% CI] |  |  |
|  |  |  |  |
| Other Cancer | N | 2598 | 1394 |
|  | Events | 73 | 40 |
|  | Person-years (*1000) | 26.27 | 13.87 |
|  | IR (%) [95% CI] | 2.78 [2.18-3.49] | 2.88 [2.06-3.93] |
|  | IR diff. [95% CI] | 0 (ref.) | 0.11 [-0.99-1.2] |
|  | Model 1 aHR [95% CI] | 1 (ref.) | 1.29 [0.84-1.97] |
|  | Model 2 aHR [95% CI] | 1 (ref.) | 1.21 [0.78-1.87] |
|  |  |  |  |
|  | | | |

EGID, eosinophilic gastrointestinal disorders distal to the esophagus. IR, incidence rate. aHR, adjusted hazard ratios.

Model 1 adjusts for age at EGID diagnosis (first biopsy), sex, county of residence, and calendar year. Model 2 adds additional adjustments for education, eczema, allergy, asthma, and eosinophilic esophagitis.

|  |  | | **Supplemental Table 10a: Mortality in EGID patients and controls with/without exposure to corticosteroids and proton pump inhibitors (End date: 31/12/2017)** | | | |
| --- | --- | --- | --- | --- | --- | --- |
|  |  | |  | | | |
|  | | Controls | EGID | EG | EGE | EC |
|  | |  | | | | |
|  | | Events/N | Events/N, aHR[95%CI] | Events/N, aHR[95%CI] | Events/N, aHR[95%CI] | Events/N, aHR[95%CI] |
| No steroid use | | 400/6551 | 104/1362, 1.11 [0.90-1.38] | 26/185, 1.77 [1.11-2.84] | 29/357, 1.53 [1.00-2.32] | 54/958, 0.86 [0.64-1.16] |
| Steroid use | | 25/494 | 7/102, 1.39 [0.61-3.15] | 1/11, 10.62 [0.32-357.16] | 2/21, 12.89 [1.06-156.04] | 5/82, 1.26 [0.43-3.66] |
| P_interaction | |  | 0.6078 | 0.3223 | 0.0983 | 0.5063 |
|  | |  |  |  |  |  |
| No PPI use | | 382/6575 | 93/1365, 1.05 [0.84-1.31] | 22/165, 1.75 [1.05-2.91] | 23/339, 1.29 [0.82-2.06] | 54/1000, 0.85 [0.63-1.15] |
| PPI use | | 43/470 | 18/99, 2.36 [1.35-4.11] | 5/31, 1.61 [0.44-5.87] | 8/39, 6.03 [2.18-16.71] | 5/40, 2.08 [0.72-6.02] |
| P_interaction | |  | 0.0079 | 0.9054 | 0.0071 | 0.1114 |

|  |  | | **Supplemental Table 10b: Risk of cancer in EGID patients and controls with/without exposure to corticosteroids and proton pump inhibitors (End date: 31/12/2016)** | | | |
| --- | --- | --- | --- | --- | --- | --- |
|  |  | |  | | | |
|  | | Controls | EGID | EG | EGE | EC |
|  | |  | | | | |
|  | | Events/N | Events/N, aHR[95%CI] | Events/N, aHR[95%CI] | Events/N, aHR[95%CI] | Events/N, aHR[95%CI] |
| No steroid use | | 209/5412 | 58/1205, 1.08 [0.81-1.44] | 8/156, 2.42 [1.02-5.71] | 12/321, 0.84 [0.45-1.55] | 40/853, 1.03 [0.72-1.46] |
| Steroid use | | 17/431 | 4/93, 1.33 [0.52-3.42] | 2/10, 12.37 [0.88-172.92] | 2/19, 1.930000e+00 [0.36-10.20] | 2/75, 0.98 [0.21-4.57] |
| P_interaction | |  | 0.6781 | 0.2488 | 0.3596 | 0.956 |
|  | |  |  |  |  |  |
| No PPI use | | 214/5460 | 56/1211, 1.05 [0.79-1.40] | 8/137, 2.67 [1.15-6.19] | 10/303, 0.73 [0.37-1.42] | 42/896, 1.02 [0.72-1.43] |
| PPI use | | 12/383 | 6/87, 3.29 [1.13-9.55] | 2/29, 3.14 [0.50-19.72] | 4/37, 3.96 [1.04-14.99] | 0/32, 0.00 [0.00-Inf] |
| P_interaction | |  | 0.0427 | 0.8756 | 0.0259 | 1 |

EC, eosinophilic colitis. EG, eosinophilic gastritis. EGE, eosinophilic gastroenteritis. EGID, eosinophilic gastrointestinal disorders distal to the esophagus. IR, incidence rate. aHR, adjusted hazard ratios. PPI, Proton-pump inhibitor

Model adjusted for age at EGID diagnosis (first biopsy), sex, county of residence, calendar year, education, eczema, allergy, asthma, and eosinophilic esophagitis.
